# Relationship between Regular Leisure-Time Physical Activity and Underweight and Overweight Status in Taiwanese Young Adults: A Cross-Sectional Study

**DOI:** 10.3390/ijerph20010284

**Published:** 2022-12-24

**Authors:** Chyi Liang, Po-Fu Lee, Ping-Chun Yeh

**Affiliations:** 1 Graduate Institute of Sport, Leisure and Hospitality Management, National Taiwan Normal University, Taipei City 106, Taiwan; 2 Department of Leisure Industry and Health Promotion, National Ilan University, Yilan County 260, Taiwan; 3 College of Humanities and Management, National Ilan University, Yilan County 260, Taiwan; 4 Exercise and Recreation Development Center, National Ilan University, Yilan County 260, Taiwan; 5 Graduate Institute of Business Administration, Fu Jen Catholic University, New Taipei City 242, Taiwan; 6 Sports Medicine Center, Fu Jen Catholic University Hospital, New Taipei City 243, Taiwan

**Keywords:** leisure-time physical activity, body mass index, obesity, young adults

## Abstract

The aim of this study was to determine the association between regular leisure-time physical activity (LTPA) and various body mass index (BMI) categories in Taiwanese young adults. A total of 10,802 young adults (18–44 years) were enrolled in a national telephone survey. The questionnaire data from this survey included socio-demographic characteristics, zip code of residence, LTPA behaviors, self-reported health status, and self-evaluated anthropometric measurements, which included height, body weight, and BMI. Regular and non-regular LTPA behaviors were defined as follows: (1) Regular LTPA: participants who reported breathing quickly and sweating when participating in 150–300 min per week of moderate-intensity LTPA or 75–150 min per week of vigorous-intensity LTPA. (2) Non-regular LTPA: the rest of the participants. The various BMI categories were defined as (1) underweight (BMI < 18.5 kg/m^2^), (2) normal weight (18.5 ≤ BMI < 24 kg/m^2^), (3) overweight (24 ≤ BMI < 27 kg/m^2^), and (4) obese (BMI ≥ 27 kg/m^2^). When compared with participants with non-regular LTPA, participants with regular LTPA exhibited lower risks of being overweight (odds ratio [OR], 0.837; 95% confidence interval [CI] 0.738–0.948) and underweight (OR, 0.732; 95% CI 0.611–0.876). However, there was no significant relationship between regular LTPA and obesity risk when using non-regular LTPA as the baseline after adjusting for potential confounders. The study results revealed that regular LTPA effectively reduced the risks of being underweight and overweight. However, for people with obesity, regular LTPA was unable to significantly decrease their obesity risk.

## 1. Introduction

Weight management is a major health issue worldwide. According to the World Health Organization, approximately 1.9 billion (39%) adults are overweight and more than 600 million (13%) are obese worldwide [1]. According to the Health Promotion Administration, Ministry of Health and Welfare, in Taiwan, underweight, normal, overweight, and obesity refer to people whose body mass index (BMI) is less than 18.5, 18.5 to 24, 24 to 30, and 30 kg/m^2^ or higher, respectively. In Taiwan, the prevalence of overweight and obesity (BMI > 24 kg/m^2^) was approximately 43.4% in the latest survey [2]. Obesity (BMI ≥ 27 kg/m^2^) can cause problems such as cardiovascular diseases, type 2 diabetes, cancer, osteoarthritis, and sleep apnea [3,4], thus compromising the quality of life.

Similarly, being underweight (BMI < 18.5 kg/m^2^) substantially increases mortality rates [5], triggers abnormal mental and physical health behavior, and lowers patients’ self-reported health [6]. A meta-analysis concluded that anorexia nervosa is associated with a mortality rate that is significantly higher than the increase caused by bulimia [7]. Being underweight may be accompanied by malnutrition, which considerably increases the risks of infection [8]. In Taiwan, approximately 3.7–8.7% of individuals are underweight [2]. Thus, weight management is crucial to lowering the social and economic burden.

Physical activity is effective for weight management. Leisure-time physical activity (LTPA) can be referred to as any non-professional and non-vocational physical activity. Increasing the amount of LTPA can reduce abdominal and visceral fat [9,10], and the amount of physical activity and fat reduction are significantly and positively correlated [11]. According to the United States National Academy of Medicine, 150 min of moderate-intensity exercise per week can effectively improve the health status of adults with obesity [12]. A lack of leisure-time physical activity is considered one of the key factors leading to overweight and obesity [13]. A longitudinal study in China also indicated that the prevalence of overweight and obesity in Chinese adults increased over seven years along with a decrease in LTPA and an increase in sedentary behavior [14]. Issues related to overweight and obesity and their associations with LTPA have been studied especially frequently. However, studies of associations between underweight and LTPA levels are sparse. A relationship between underweight prevalence and lower levels of physical activity among male youth was observed [15], but non-significant associations were found in a study by Pengpid et al. among adults in Laos [16]. Therefore, whether LTPA is equally effective in improving the health of people who are underweight and whether LTPA can mitigate the risk factors for overweight and obesity and being underweight remain uncertain. The aim of this study was to determine the association between regular leisure-time physical activity and various BMI categories in Taiwanese young adults.

## 2. Materials and Methods

### 2.1. Study Sample and Data-Collection Procedures

Cross-sectional study data were obtained from a nationwide survey of children and adolescents (13–17 years), adults (18–64 years), and older adults (65 years and older), known as the Taiwan National Physical Activity Survey (TNPAS), conducted by the Sports Administration, Ministry of Education, in Taiwan. Recruitment was by random-digit-dialing using proportional-stratified sampling with multiple factors (e.g., age, gender, and geographic districts). The reliability of the research method and detailed procedure has been described elsewhere [17]. Citizens aged over 13 years and stratified by 22 cities/counties across the country were selected to become the sample population. The sample size of each city/county was determined by its proportion of the national population in Taiwan. The total sample size was 25,526 in 2020, with sampling errors of 3–5% and a confidence interval (CI) of 95%, which allowed for a sufficient sample size and statistical power to be obtained. Subsequently, a telephone interview (CATI) was conducted using a computer-assisted system from August to October in 2020. To ensure the quality of data collection, a group of well-trained and experienced interviewers was employed for the CATI. The following data were collected through the telephone survey: socio-demographic characteristics (i.e., age, gender, education, and occupation), physical activity behaviors, self-reported health status, self-evaluations (e.g., body height and body weight), and zip code of residence. Finally, since we were focused on Taiwanese younger adults, a total of 10,802 young adults (18–44 years) were selected for this study. The participants were fully informed about the objective, procedures, and content of the study. The study was conducted according to the guidelines of the Declaration of Helsinki and all procedures were approved by the Institutional Review Board of the Fu Jen Catholic University in Taiwan (FJU- IRB C109085). Oral consent was given before the interview. All relevant information was contained in the de-identified secondary dataset and was released for public research purposes.

### 2.2. Data Collection

Multiple demographic characteristics of participants were recorded in this study, including age, gender, education, occupation, and self-reported health status. The age of participants was divided into 18–24, 25–29, 30–34, 35–39, and 40–44 years; education was categorized as elementary school or lower, junior or senior high school, and college or higher. Detailed categories of occupation included white collar, government servant, blue-collar, owner/manager, specialists, student, housewife, retired, freelancer, jobless, and other. The self-reported health status was excellent or good, fair, and very bad or poor.

### 2.3. Self-Reported Anthropometrics and Obesity Status

Both body height and weight were considered as self-reported anthropometric variables that were used to calculate BMI (kg/m^2^). In this study, BMI values were calculated from self-reported height and weight provided by respondents. BMI is weight (kg) divided by height (in meters) squared (m^2^). The obesity status was defined according to the following categories from the Taiwan Ministry of Health and Welfare, Health Promotion Administration: (1) underweight (BMI < 18.5 kg/m^2^), (2) normal weight (18.5 ≤ BMI < 24 kg/m^2^), (3) overweight (24 ≤ BMI < 27 kg/m^2^), and (4) obese (BMI ≥ 27 kg/m^2^) [18].

### 2.4. LTPA Assessment

A series of questions presented through a CATI was utilized to determine either regular or non-regular LTPA. The CATI process was as follows: First, the participant’s current LTPA was assessed by the following question, “Have you taken part in any LTPA in the past month?” Second, both the frequency and duration of LTPA participation were assessed by the following questions when the respondent provided a positive response: “How many times do you participate in LTPA per week?”, and “How many minutes do you usually spend at one time?” Third, the LTPA intensity was assessed by the description of breathing and sweating status from the participant, and those questions included “When you are doing LTPA, you usually feel…”, and then, the following structural answers were selected and described by the respondent, “No changes in my breath and sweating,” “I breathe faster but do not sweat,” “I breathe normally but sweat,” “I breathe quickly and sweat.” When the respondent indicated they usually breathed quickly and sweated, they were considered to engage in moderate-intensity LTPA. Finally, regular and non-regular LTPA groups were defined by the following conditions: (1) Regular LTPA group: participants who reported breathing quickly and sweating during participation in 150–300 min per week of moderate-intensity LTPA or 75–150 min per week of vigorous-intensity LTPA. (2) Non-regular LTPA group: the rest of the participants.

### 2.5. Statistical Analysis

SAS 9.4 (SAS Institute, Cary, NC, USA) was utilized to perform multiple statistical analyses, including Student’s *t*-tests, chi-square tests, and multiple linear regression analysis. Continuous variables were analyzed using the Student’s *t*-test, and categorical variables were analyzed using the chi-square test. BMI was a dependent variable used to examine the relationship between regular LTPA and BMI after adjustment for potential confounders using multiple linear regression analysis. Adjusted odds ratios (ORs) with 95% confidence intervals (CIs) were applied to obese, overweight, or underweight, calculated from unconditional logistic regression models based on regular LTPA. In this study, test values were presented as means ± standard deviation (SD) or frequency percentages. The test results were evaluated with two-tailed tests and were defined as statistically significant at *p* < 0.05.

## 3. Results

Table 1 shows the demographic characteristics of the participants. The 10,802 data points were dichotomized into groups with respect to leisure-time physical activity (LTPA) status; most of the participants (76%) were classified into the non-regular LTPA group. Except for height and BMI, significant differences were found between groups (*p* < 0.05) on all relevant variables, including age, gender, body weight, obese status, education, occupation, and self-reported health status. Among participants in the non-regular LTPA group, the rates of underweight (9.00%), overweight (19.50%), and obese (15.00%) were higher than in the regular LTPA group and 18% of these participants said that their health status was very bad or poor, which was also higher than in the regular LTPA group (11.00%).

Table 2 indicates the result of the multivariate regression of regular LTPA to BMI score. There was a positive relationship between regular LTPA and BMI scores (*β* = 0.014, *p* < 0.05); the more regular LTPA adults engaged in, the lower their BMI score. However, adjusted for age, gender, self-reported health status, occupation, and education, the explanatory power decreased (*β* = 0.005). The results of the multivariate logistic regression for regular LTPA to obesity status (obesity, overweight, underweight) are shown in Table 3. In model 2, when using non-regular LTPA as the baseline, participants in the regular LTPA group exhibited lower risks of overweight and underweight (OR, 0.837; 95% CI 0.738–0.948, OR, 0.732; 95% CI 0.611–0.876). However, there was no significant relationship between regular LTPA and obesity risk when using non-regular LTPA as the baseline in model 2.

## 4. Discussion

Based on the suggestions made by the American College of Sports Medicine (ACSM), this study defined ≥150 min of moderate-intensity exercise or ≥75–150 min of high-intensity exercise per week as representing a habit of exercising regularly or having non-regular LTPA. The study results revealed that regular LTPA effectively reduced the risks of underweight and overweight. However, for people with obesity, regular LTPA was unable to significantly decrease their obesity risk.

According to the study results, regular LTPA reduced the odds of being underweight by 26.8%. The causes of being underweight include anorexia nervosa, behavior [19], and picky eating [20], which result in insufficient calorie intake for extended periods of time. Studies have indicated that 120 min of aerobic exercise per week can effectively increase the weight and fitness of patients who are underweight [21]. For patients with anorexia nervosa, exercise intervention can effectively improve their nutritional status [22] and quality of life [23]. Additionally, exercise intervention can improve their weight and eating disorder behaviors [24] considerably more than that achieved with traditional cognitive-behavioral therapy. Although this study did not explore the specific causes of becoming underweight, the multivariate logistic regression analysis (adjusted for variables such as age, gender, self-reported health status, occupation, and education) showed that exercise effectively decreased the odds of being underweight, suggesting that developing regular exercise habits may be key to lowering the risk of being underweight.

The current study indicated that LTPA can effectively diminish the odds of being overweight. LTPA can successfully reduce abdominal and visceral fat [9,10], and the amount of physical activity and fat reduction are significantly and positively correlated [11]. Sedentary lifestyle and minimal physical activity are the main causes of increasing obesity prevalence [25]. In the United Kingdom, decreased physical activity level has been found to be the primary cause of high obesity prevalence [26]. Consistently, the present study found that regular LTPA can effectively lower the odds of being overweight.

Although LTPA can reduce the odds of being overweight considerably, it was unable to lower the odds of obesity; this may be a consequence of the definition of LTPA. The ACSM asserted that 150 min of moderate-intensity exercise per week can effectively maintain people’s weight and health status. However, 150–200 min of moderate-intensity exercise per week can only achieve a minimal reduction in body weight. Without dietary intervention, >250 min of moderate-intensity exercise per week is required to have any pronounced effect [12]. Other review-based studies have also indicated that 150 min of moderate-intensity exercise is ineffective in reducing weight [27] and have recommended 150–300 min of moderate-intensity or 75–150 min of high-intensity exercise per week [28] for weight loss. Therefore, for patients with obesity, exercise intervention may be insufficient, and dietary intervention or cognitive-behavioral therapy may be necessary.

Although this study incorporated various intervening variables, it still had the following limitations: The main limitation was the cross-sectional design. This study hypothesized an association between LTPA and BMI in young Taiwanese adults. However, the effect of LTPA on BMI is not immediate. Therefore, longitudinal study designs can reveal whether individual BMI values vary with or without LTPA participation. Additionally, an individual’s BMI may also influence an individual’s participation in LTPA. Therefore, the causal relationship between LTPA and BMI needs to be further verified. The second limitation was the consequence for analysis of the low proportion of study participants with underweight (8.53%). However, this study had a sufficient sample size, similar to those of previous studies on physical activity and BMI categories [28,29,30,31] and used questionnaires with favorable reliability and validity, which have also been used in other studies [30]; thus, the sample size, study reliability, and study validity were representative of the overall population. Moreover, the data were limited to Taiwan. Future studies should investigate other ethnic groups, countries, and people of different financial status. Finally, factors other than those covered in the questionnaires may have caused the participants to be underweight or overweight/obese, which were not examined in this study. Future studies should include other factors, such as dietary habits, energy intake, and nutritional intake, particularly for people who are underweight.

## 5. Conclusions

In summary, this study found that regular LTPA (150–300 min per week of moderate-intensity LTPA or 75–150 min per week of vigorous-intensity LTPA) effectively reduced the risks of being underweight and overweight. However, for people with obesity, regular LTPA was unable to significantly decrease their obesity risk. Future studies should investigate the independent or interactive effects of a combined approach of physical activity and dietary control on body composition development.

## Figures and Tables

**Table 1:** Demographic characteristics.

Variables	LTPA Status		*t*-Value or *x*^2^	*p*-Value
	Regular LTPA (*n* = 2592)	Non-Regular LTPA (*n* = 8210)		
**Age (y)** ^a^			2847.81	<0.001 *
18–24	809 (31.20%)	1598 (19.50%)		
25–29	480 (18.50%)	1412 (17.20%)		
30–34	443 (17.10%)	1429 (17.40%)		
35–39	403 (15.50%)	1880 (22.90%)		
40–44	457 (17.60%)	1891 (23.00%)		
Gender (% men) ^a^	1698 (65.5%)	4001 (48.7%)	68.79	<0.001 *
Height (cm) ^b^	169.26 ± 8.68	165.75 ± 8.31	–1.17	0.244
Body weight (kg) ^b^	66.49 ± 13.26	63.80 ± 14.07	–5.71	<0.001 *
BMI (kg/m^2^) ^b^	23.08 ± 3.55	23.07 ± 4.05	0.90	0.960
Obese Status (%) ^a^			141.84	<0.001 *
Underweight	184 (7.10%)	737 (9.00%)		
Normal weight	1475 (56.90%)	4285 (52.20%)		
Overweight	485 (18.70%)	1598 (19.50%)		
Obese	353 (13.60%)	1230 (15.00%)		
Education (%) ^a^			306.70	<0.001 *
Elementary school or lower	2 (0.10%)	30 (0.40%)		
Junior or senior school	553 (21.40%)	2547 (31.10%)		
College or higher	2035 (78.60%)	5608 (68.50%)		
Occupation (%) ^a^			1932.19	<0.001 *
White collar	588 (22.80%)	2033 (24.90%)		
Government servant	228 (8.80%)	497 (6.10%)		
Blue collar	380 (14.70%)	1970 (24.10%)		
Owner/manager	139 (5.40%)	337 (4.10%)		
Specialists	289 (11.20%)	812 (10.00%)		
Student	616 (23.90%)	895 (11.00%)		
Housewife	80 (3.10%)	641 (7.90%)		
Retired	1 (0.00%)	13 (0.20%)		
Free-lancer	104 (4.00%)	248 (3.00%)		
Jobless	119 (4.60%)	636 (7.80%)		
Other	36 (1.40%)	78 (1.00%)		
Self-reported health status (%) ^a^			257.64	<0.001 *
Excellent or good	2185 (85.00%)	6071 (75.50%)		
Fair	103 (4.00%)	520 (6.50%)		
Very bad or poor	282 (11.00%)	1449 (18.00%)		

Abbreviations: BMI, body mass index; LTPA, leisure-time physical activity. * *p* < 0.05. ^a^ Values expressed as *n* (percentage); ^b^ Values expressed as mean ± standard deviation.

**Table 2:** Multivariate regression for regular LTPA to BMI score.

Variables	Model 1 (Unadjusted)			Model 2 (Adjusted ^a^)		
	*β*	SE	*p*-Value	*β*	SE	*p*-Value
Regular LTPA	0.014	0.078	0.172	0.005	0.074	0.588
Non-regular LTPA	Ref.	-	-	Ref.	-	-

Abbreviations: LTPA, leisure-time physical activity; SE, standard error. * *p* < 0.05. **^a^** Adjusted for age, gender, self-reported health status, occupation, and education.

**Table 3:** Multivariate logistic regression for regular LTPA to underweight, overweight, and obesity risk.

Variables	Model 1 (Unadjusted)				Model 2 (Adjusted ^a^)			
	OR	95% CI	*p*-Value		OR	95% CI	*p*-Value	
**Underweight**								
Regular LTPA	0.726	0.611–0.862	<0.001 *		0.732	0.611–0.876	0.001 *	
Non-regular LTPA	Ref.	-	-		Ref.	-	-	
Overweight								
Regular LTPA	0.881	0.783–0.991	0.035 *		0.837	0.738–0.948	0.005 *	
Non-regular LTPA	Ref.	-	-		Ref.	-	-	
Obesity								
Regular LTPA	0.832	0.729–0.950	0.007 *		0.880	0.759–1.020	0.090	
Non-regular LTPA	Ref.	-	-		Ref.	-	-	

Abbreviations: CI, confidence interval; LTPA, leisure-time physical activity; OR, odds ratio. * *p* < 0.05. ^a^ Adjusted for age, gender, self-reported health status, occupation, and education.

## Data Availability

The raw data are not publicly available due to ethical restrictions.
